# Supplementary material for: Realistic and complex visual chasing behaviors trigger the perception of intentionality
Source: PLoS One. 2023 Apr 14;18(4):e0284485. doi: 10.1371/journal.pone.0284485 (PMC10104337; doi:10.1371/journal.pone.0284485)
Supplement: Attachment — Submitted filename: ResponseLetter.docx [file pone.0284485.s002.docx]

Response to Reviewers

We would like to again thank the reviewer’s time and effort to comment on the manuscript and edited the manuscript to address their concerns. Below you can find responses to each concern raised by the reviewers.

Reviewer #1: I am not sure if the authors uploaded an earlier manuscript given that they have said that effect sizes have now been provided. They have not been provided and the Bayes factors still appear as though they are being used as an effect size for the frequentist statistics. Given that frequentist and Bayesian philosophies are fundamentally different and based on different assumptions this is inappropriate.

An appropriate way of following up null results in frequentist statistics is equivalence testing:
Lakens, D., Scheel, A. M., & Isager, P. M. (2018). Equivalence Testing for Psychological Research: A Tutorial. Advances in Methods and Practices in Psychological Science, 1(2), 259–269. <https://doi.org/10.1177/2515245918770963>

Where I can accept that the authors may wish to provide both Frequentist and Bayesian statistics they should be clearly separated so one is not used as an effect size for the other. I believe that potentially my review was misunderstood.

Here is a primer on effect sizes for frequentist statistics:
Lakens, D. (2013). Calculating and reporting effect sizes to facilitate cumulative science: A practical primer for t-tests and ANOVAs. Frontiers in Psychology, 4. <https://doi.org/10.3389/fpsyg.2013.00863>

We apologize for the misunderstanding. For our revision, we uploaded a new manuscript version corresponding to the changes described in our revision letter, but did not delete the original manuscript (given the way thet submission portal is set up, it wasn’t clear how the system wanted us to do version control when building the pdf for reviewers). We have now removed the previous submissions and only kept the most recent revised manuscript (both with track changes and without). We agree it is inappropriate to treat Bayes’ Factors as an effect size for frequentist statistics and these are clearly indicated in the revised manuscript.

We would like to thank the reviewers for evaluating our manuscript. We have tried to address all the reviewers’ concerns in a proper way and believe that our paper has improved considerably. We would be happy to make further corrections if necessary and look forward to hearing from you soon.
